# Secular trend of non-communicable chronic disease prevalence throughout the life span who endured Chinese Great Famine (1959–1961)

**DOI:** 10.1186/s12889-023-16142-4

**Published:** 2023-06-26

**Authors:** Xiaoxue He, Xiaojuan Shi, Degong Pan, Huihui Wang, Xue Zhang, Lining Pu, Mingxiu Luo, Jiangping Li

**Affiliations:** 1 Department of Epidemiology and Health Statistics, School of Public Health, Ningxia Medical University, Yinchuan, 750004 Ningxia Hui Autonomous Region China; 2 Department of Biochemistry and Molecular Biology, School of Basic Medical Sciences, Ningxia Medical University, Yinchuan, 75004 Ningxia China; 3 Key Laboratory of Environmental Factors and Chronic Disease Control, Ningxia Medical University, Yinchuan, 750004 Ningxia, Hui Autonomous Region China

**Keywords:** Famine, Non-communicable chronic diseases, Age-period-cohort models, Prevalence, China Family Panel Studies

## Abstract

**Background:**

Famine is a risk factor for non-communicable chronic diseases (NCDs), which account for over 80% of deaths in China. The effect of famine on the prevalence of NCDs in terms of various age groups, time periods and cohorts is currently poorly understood.

**Objective:**

This study aims to explore long-term trends in the impact of China’s Great Famine (1959–1961) on NCDs in China.

**Methods:**

This study used data from the 2010–2020 China Family Panel Longitudinal Survey across 25 provinces in China. The subjects were aged 18–85 years, and the total number of subjects was 174,894. The prevalence of NCDs was derived from the China Family Panel Studies database (CFPS). An age-period-cohort (APC) model was used to estimate the age, period and cohort effects of NCDs in 2010–2020 and the effect of famine on the risk of NCDs in terms of cohort effects.

**Results:**

The prevalence of NCDs increased with age. Additionally, the prevalence did not clearly decrease over the survey period. Regarding the cohort effect, people born in the years adjacent to the famine period had a higher risk of NCDs; additionally, females, those born in rural areas, and those who lived in provinces with severe famine and post-famine had a higher likelihood of NCDs.

**Conclusions:**

Experiencing famine at an early age or the experience of famine in a close relative’s generation (births after the onset of famine) are associated with an increased risk of NCDs. Additionally, more severe famine is associated with a higher risk of NCDs.

**Supplementary Information:**

The online version contains supplementary material available at 10.1186/s12889-023-16142-4.

## Introduction

China has the largest ageing society in the world, with the number of people aged 60 and over already reaching 264 million in 2020, accounting for 18.7%, and expected to increase to 28% in 2040 [1, 2]. Aging population increasing the risk and burden of non-communicable diseases (NCDs) and posing a major challenge to the public health services [1]. NCDs, which are characterized by high morbidity, disability and mortality, are the leading cause of death worldwide [3]. In the World Health Statistics 2022 report, the World Health Organization (WHO) noted that the global proportion of deaths attributable to NCDs increased from almost 60.8% in 2000 to 73.6% in 2019 [4, 5]. Previous studies have shown that NCDs are mostly influenced by personal factors that can be prevented and improved during the life cycle, such as an unhealthy diet, physical inactivity, obesity, smoking and excessive alcohol consumption [5–10]. Furthermore, for the entire population, NCDs are also affected by economic, social, ecological and early life events, of which famine is one key factor that requires further research attention [11].

Famine is defined as an acute onset of extreme starvation that leads to excess mortality and reduced fertility [12, 13]. Famine may lead to weight loss, growth retardation and malnutrition [14]. Indeed, famine even causes premature death and has a serious impact on mortality [11, 14], which cannot be predicted or changed. In addition, early evidence suggests that prenatal malnutrition and childhood famine exposure are associated with an increased risk of NCDs [15–21]. Between the spring of 1959 and the end of 1961, China experienced one of the worst famines in its history, resulting in the premature death of tens of millions [22]. Those who experienced famine and were born during the famine are now aging and face NCDs that required more attention. Most of the studies recently mainly explore the association between famine exposure and single NCDs. Exploring the effects can provide a valid basis for healthy development for older adults in ageing time.

## Methods

### Data source

The data for this study were sourced from the China Family Panel Studies (CFPS) database, a national, large-scale, multidisciplinary social tracking survey project. These data reflect changes in China’s society, economy, population, education and health. The CFPS database is implemented by the Institute of Social Science Survey (ISSS) of Peking University, and it provides a database for academic research and public policy analysis. In the CFPS, computer-aided survey technology is used to conduct the interviews with the participants in order to meet diverse design needs, improve the access efficiency and ensure the data quality. The CFPS sample covers 25 provinces/municipalities/autonomous regions, with good regional representation [23]. Ethical approval was granted by the Biomedical Ethics Committee of Peking University. The CFPS database is a biennial survey, and this study used data from the survey years 2010, 2012, 2014, 2016, 2018 and 2020 for the population aged 18–85 years.

### Statistical analyses

According to the requirements of the APC model, birth cohort = period − age needs to be satisfied; the famine lasted for a 3 year period from 1959–1961, so the study period was arranged in 3 year intervals. As the CFPS database is a biennial follow-up study, we used the calculated NCD prevalence rates for 2012, 2014, 2018 and 2020 as reasonable estimates for the NCD prevalence in 2013 and 2019 to obtain data for survey years 2010, 2013, 2016 and 2019. The ages of the participants ranged from 18 to 85 years, and they were divided into 21 consecutive age intervals(19–21,22–25…82–84), with 18 and 85 years being placed in the first and last age groups. The birth cohorts comprised 23 consecutive 3 year cohort intervals from 1925 to 2002. The 2013 survey year and 1948 birth cohorts were specified as the reference groups for the period and cohort effects, and the relative risk ratios for the period and cohort relative to the respective reference groups for the disease were calculated separately. We used the age-period-cohort web tool (Age Period Cohort Analysis Tool [cancer.gov]) [24–26] provided by the National Cancer Institute for the parameter estimation and analysis [26].

Males versus females, individuals living in urban versus rural areas, and individuals from different provinces experienced varying famine intensity [27–30]. We defined seven provinces as experiencing severe famine exposure (Sichuan, Anhui, Guizhou, Qinghai, Gansu, Guangxi, Henan), and the other provinces were defined as experiencing mild famine [22]. Firstly, we examined the trends in the prevalence of NCDs in China by gender, urban versus rural areas, and provinces with different famine intensities from 2010 to 2020. The age, period and cohort effects in the APC model were then assessed using the APC framework.

## Results

### Trends in NCD Prevalence

**Fig. 1:**
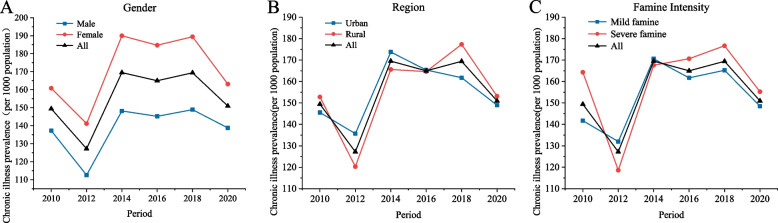
Trends in the prevalence of NCDs by (**A**) gender, (**B**) rural versus urban areas and (**C**) famine intensity

The average prevalence of NCDs among individuals aged 18–85 years surveyed in the CFPS from 2010 to 2020 was 155.4 per 1,000 people (Table 1*, *Fig. 1). For both genders, the NCD prevalence was lowest in 2012 and highest in 2014, and there was a higher prevalence among females than males in all the survey periods (Fig. 1A). There were no major differences in the prevalence of NCDs in urban versus rural areas within each survey period, and 2012 showed the lowest NCD prevalence across urban and rural areas (Fig. 1B). Additionally, a similar pattern was observed in terms of the provinces with different famine intensities (Fig. 1C).

**Table 1:** Incidence of NCDs by group classification, 2010–2020

Group/Year	2010 (n‰)		2012 (n‰)		2014 (n‰)		2016 (n‰)		2018 (n‰)		2020 (n‰)		Total (n‰)	
Male	2148	(137.2)	1664	(112.5)	2182	(148.2)	2309	(145.2)	2138	(148.9)	1478	(138.8)	11,919	(138.5)
Female	2683	(160.8)	2195	(141.2)	2913	(190.0)	2941	(184.7)	2781	(189.4)	1739	(163.1)	15,252	(171.7)
Urban	2191	(145.5)	1852	(135.7)	2503	(173.7)	2595	(165.4)	2390	(161.8)	1657	(148.9)	13,188	(155.7)
Rural	2640	(152.8)	2007	(120.3)	2592	(165.6)	2655	(164.6)	2529	(177.3)	1560	(153.1)	13,983	(155.0)
Severe famine provinces	1805	(164.3)	1282	(118.6)	1798	(167.6)	2001	(170.6)	1874	(176.7)	1204	(155.2)	9964	(159.1)
Mild famine provinces	3026	(141.7)	2577	(132.0)	3297	(170.6)	3249	(161.7)	3045	(165.2)	2013	(148.5)	17,207	(153.2)
All	4831	(149.4)	3859	(127.2)	5095	(169.5)	5250	(165.0)	4919	(169.4)	3217	(150.9)	27,171	(155.4)

n‰ is the number of NCDs per 1,000 individuals in the population

### Age-Specific Prevalence Rates for NCDs

**Fig. 2:**
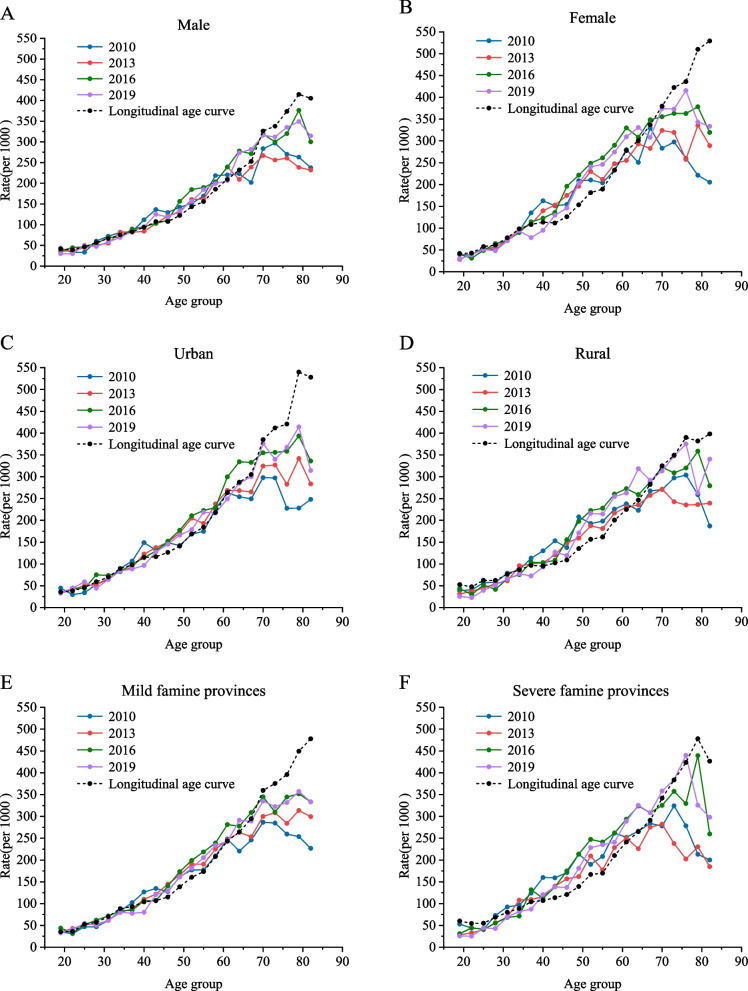
Prevalence of NCDs by age for (**A**) male and (**B**) female gender, (**C**) urban versus (**D**) rural areas and (**E**) provinces with mild versus (**F**) severe famine intensity, 2010–2019

**Fig. 3:**
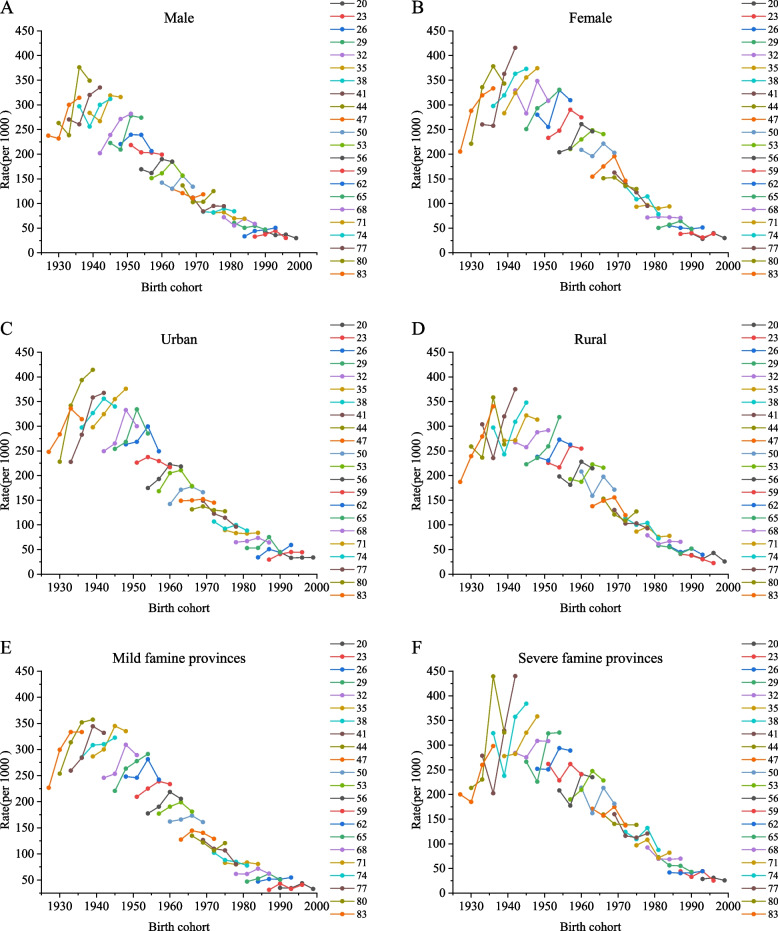
Prevalence of NCDs by age-group cohort for (**A**) male and (**B**) female gender, (**C**) urban versus (**D**) rural areas and provinces with (**E**) mild versus (**F**) severe famine intensity, 2010–2019

For our study, the population data and NCD prevalence rates for 2013 and 2019 were feasibly estimated, resulting in survey data in 3 year intervals for 2010, 2013, 2016 and 2019. The trends in NCD prevalence from 2010 to 2019 showed stable trends by gender, with the largest differences in males between 2013 and 2016 and a slight decrease in 2019 for females (SI Appendix Table S1, Fig. 2A and B). The prevalence of NCDs in urban areas decreased slightly in 2019, while the prevalence in rural areas showed a gradual increase after the lowest point in 2013 (Fig. 2C and D). There was an overall increasing trend in the prevalence of NCDs over time across the provinces with different famine intensities d (Fig. 2E and F). In terms of the NCD prevalence rates in each cohort by age group, there was a decreasing trend with more recent birth cohorts there was a decreasing trend t, thus indicating a lower prevalence of NCDs in the more recent birth cohorts (SI Appendix Table S2, Fig. 3).

### Age-Period-Cohort Effects on NCD Prevalence

**Fig. 4:**
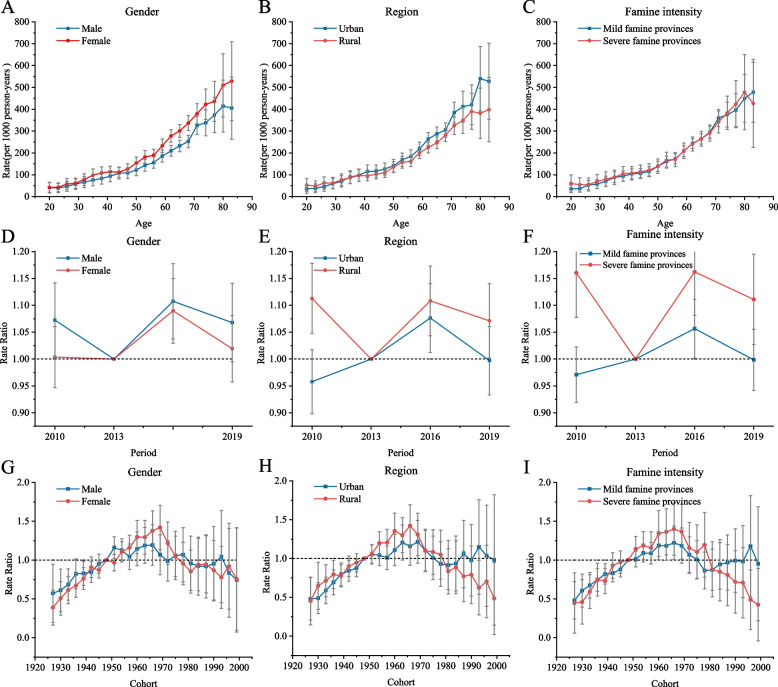
Parameter estimates of the age effects on NCD prevalence by (**A**) gender, (**B**) rural versus urban regions and (**C**) provinces with different famine intensities; parameter estimates of the period effects on NCD prevalence by (**D**) gender, (**E**) rural versus urban regions and (**F**) provinces with different famine intensities; and parameter estimates of the cohort effects on NCD prevalence by (**G**) gender, (**H**) rural versus urban regions and (**I**) provinces with different famine intensities, 2010–2019. Abbreviation: APC = age-period-cohort

In the APC model, the longitudinal age curve is used to assess the age effects. The age trends in the prevalence of NCDs were similar throughout the study period, indicating that NCD prevalence increases with age (Fig. 4). In terms of gender, the prevalence of NCDs was higher in male at all ages (Fig. 4A). Furthermore, in those aged 35 years and above, there was a higher prevalence of NCDs in urban areas (Fig. 4B). In addition, the difference in prevalence across age groups was small between provinces exposed to severe versus mild famine, but the overall prevalence was higher in provinces with severe famine (Fig. 4C).

Across genders, the prevalence of NCDs did not improve throughout the study period (Fig. 4D). As shown in the figure, the trend in NCD prevalence based on period was similar in urban and rural areas and in provinces with different famine intensities (Figs. 4E, 4F). The NCD prevalence did not improve significantly in any of the survey years except for in urban areas and provinces with mild famine exposure in 2010.

In terms of the cohort effect, the NCD prevalence followed an inverted U-shaped distribution across the cohorts in all three groups (gender, urban versus rural areas and province famine intensity; (Fig. 4G–I). Females born during and around the famine had a higher risk of NCDs (Fig. 4G). Additionally, those living in rural areas (Fig. 4H) and in provinces with severe famine exposure had a higher risk of NCDs.

## Discussion

Our findings highlight the effect of the 1959–1961 Great China Famine on NCDs. Specifically, pre-birth famine exposure was associated with an increased risk of NCDs in later life. By combining longitudinal survey data with APC models to analyze trends in the prevalence of NCDs, we found similarities in prevalence across these three important time segments (age, period and cohort). The age effects indicated that the prevalence of NCDs increased with age. The period effect showed that the risk of NCDs varied between the survey times, but these differences were not significant. In terms of cohort effects, the risk was higher among those who were born during, before and soon after the famine.

The results regarding the age effect, which is the effect of age on disease prevalence, were similar to those of previous studies, as the prevalence of NCDs was highest among older people, regardless of gender, urban versus rural regions, or famine intensity in the province [31–33]. Similarly, the results in terms of gender were consistent with previous research showing a slightly higher prevalence of NCDs in females than males [34]. The higher prevalence of NCDs in urban areas observed in this study may be related to the increased health risk factors associated with urbanization, such as changes in dietary patterns and environmental pollution [27, 34, 35]. Finally, the age effects on NCD prevalence were similar across provinces with different intensities of famine exposure.

Period effects refer to changes in disease rates in populations affected by human or social factors. From the results, it is clear that the prevalence of NCDs did not improve throughout the survey period. It is likely that the risk of NCDs continues to rise due to the increased health risk factors resulting from the ageing population and rapid urbanization [35]. During the survey period, males had a higher risk of NCDs, possibly because males had more risk factors for NCDs, such as smoking and drinking [36]. Moreover, the speed of ageing in rural areas has been faster than in urban areas in recent years, and rural people are at a disadvantage in terms of social economy, medical resources and other aspects [27]. As such, people in rural areas are more susceptible to NCDs. The extreme similarities in NCD prevalence between different provinces and urban and rural areas suggests a greater risk of NCDs in provinces where the intensity of famine and rural was high during the survey period, which may be due to the health lag caused by famine [15–18, 37].

Cohort effects refer to changes in disease prevalence due to different levels of exposure to risk factors across generations; in this one, one notable finding was the high risk of NCDs in cohorts born during and around the famine and post-famine (This group is defined as those born after the famine and whose risk of chronic disease is referenced to the 1948 birth cohort). Studies have claimed that the adverse effects of famine on survivors mean they are more at risk of NCDs [38, 39]. Other studies have demonstrated that severe famine within 3 years can affect the health of at least two generations [40, 41]. Additionally, gender discrepancies have been found, with one study reporting that the mortality rate for girls was around 7% higher than that of boys during the famine, due to Chinese traditional cultural beliefs, more care for male babies and thus more opportunities for survival [28–30, 42]. Other explanations about the starvation exposure can lead to NCDs in females is the effects of genetic changes triggered by unfavorable prenatal environments that also vary by gender [43, 44]. In addition, rural areas were more affected by the famine due to severe food supply shortages and restrictions on rural–urban migration during the famine [22, 30, 45]. And seven provinces, including Sichuan, Anhui, Guizhou, Qinghai, Gansu, Guangxi and Henan, suffered greater loss of life during the famine compared to other provinces [11, 46]. The main reason for these large differences in famine intensity was that the implementation of the central government’s grain quota procurement differed across the provinces [22]. This is similar to the findings of some studies, such as the increased prevalence of diabetes among those born in more severely affected areas compared to less affected famine areas, and the same results may exist for other NCDs [18, 47].

The risk of NCDs varies significantly between males and females, urban and rural, and between groups of provinces with different famine intensities, these severity patterns may be the reason for the significant differences in the risk of NCDs. Indeed, those born in 1947–1961 were exposed to the famine during gestation, as infants, and as school children (0–12 years), and their high risk of NCDs may be due to their experience of famine [12, 16, 17, 46]. Furthermore, although those born in 1962–1978 did not experience famine themselves, parental exposure to famine prior to pregnancy. Some studies show that the children of hungry mothers are more likely to have health problems [18, 48, 49]. Maternal malnutrition or malnutrition in prenatal could exacerbates the risk of developing certain NCDs, reinforcing the source of the increased risk of NCDs prevalence in the post-famine birth cohort in the context of cumulative exposure [12, 18, 22, 31, 37, 50]. In addition, there are sibling-designed studies used to exclude and detect confounding factors, and the results suggest that individual epigenetic differences between famine-affected and non-fame-affected same-sex siblings in middle and old age, such as changes in methylation levels [18, 51]. Overall, the high risk of NCDs among this cohort may be associated with their parents’ experience of famine, which may subsequently affect the individuals throughout their life span [31, 52–54].

The latest report shows that the goal of eradicating hunger and malnutrition by 2030 is not expected to be achieved, and that 8% of the world's population will still face starvation [55]. Unhealthy diets have long been associated with increasing rates of NCDs globally, but the burden of disease from food shortages and famine is often underestimated [56]. As the problem of famine and malnutrition remains unsolved to date, we should be concerned about the future incidence of NCDs in famine-exposed populations and the burden of disease that it brings, in addition to the outcome that it leads to death. In addition, aging population is increasing in China and globally, and the consequent health problems should be focused that have experienced famine, with an emphasis on increased screening, treatment and management of diseases and better social protection.

Currently, few studies have used the APC model to explore the effect of famine exposure on the prevalence of NCDs. In our study, we examined the effects of age, period and cohort on the risk of NCDs and, thus, identified priority groups for prevention and treatment. The limitations of our study are as follows [1]. The database lacks migration data for people who experienced famine in the early years, and it was, thus, not possible to determine their demographic information at the time of the famine; as a result, some people living in urban or rural areas or certain provinces during the survey period may not have lived in those areas during the famine period [2]. Since the time span of the famine was 3 years and the CFPS database is followed up every 2 years, in order to meet the conditions of APC, we used the survey data from 2012 and 2014 to obtain the average prevalence to estimate the results of the 2013 survey year, and we used the survey data of 2018 and 2020 to obtain the average prevalence to estimate the results of the 2019 survey year; although all the data during the CFPS survey period were used, there may have been deviations among the years that were not accounted for by these estimates [3]. Famine is a natural event, and the population selected bias for this study is an unavoidable.

## Conclusions

Famine exposure is associated with an increased risk of NCDs. The age effect showed that the prevalence of NCDs increased with age, the period effect showed that the prevalence differed across the survey period, and the cohort effect showed that those born during and around the time of the famine were at higher risk of NCDs. Across the population, groups that suffered more severely from famine, including females and those in rural areas and provinces with severe famine exposure, were at higher risk of NCDs. In conclusion, preventing famine and actively improving the nutritional status of females of childbearing age are important actions for reducing NCDs.

## Supplementary Information

**Additional file 1: Table S1.** Prevalence of NCDs by age for male and female gender, urban versus rural areas and provinces with mild versus severe famine intensity, 2010–2019. **Table S2.** Prevalence of NCDs by age, period, and birth cohort, 2010-2019 (per 1000 population).

## Data Availability

Publicly available datasets were analyzed in this study. This data can be found at: https://opendata.pku.edu.cn.
