# Supplementary material for: Secular trend of non-communicable chronic disease prevalence throughout the life span who endured Chinese Great Famine (1959–1961)
Source: BMC Public Health. 2023 Jun 26;23:1238. doi: 10.1186/s12889-023-16142-4 (PMC10294512; doi:10.1186/s12889-023-16142-4)
Supplement: Supplementary file 1 — Additional file 1: Table S1. Prevalence of NCDs by age for male and female gender, urban versus rural areas and provinces with mild versus severe famine intensity, 2010–2019. Table S2. Prevalence of NCDs by age, period, and birth cohort, 2010-2019 (per 1000 population). [file 12889_2023_16142_MOESM1_ESM.docx]

TableS1：Prevalence of NCDs by age for male and female gender, urban versus rural areas and provinces with mild versus severe famine intensity, 2010–2019

| Gender | Male(n%) | | | | | | | | Female(n%) | | | | | | | |
| --- | --- | --- | --- | --- | --- | --- | --- | --- | --- | --- | --- | --- | --- | --- | --- | --- |
| Age/Period | 2010 | | 2013 | | 2016 | | 2019 | | 2010 | | 2013 | | 2016 | | 2019 | |
| 18-21 | 38 | (4.3) | 28 | (3.6) | 30 | (3.7) | 19 | (3.0) | 38 | (4.0) | 23 | (2.8) | 34 | (4.0) | 18 | (3.0) |
| 22-24 | 23 | (3.3) | 27 | (3.7) | 31 | (4.5) | 15 | (3.0) | 31 | (3.8) | 35 | (4.0) | 24 | (3.1) | 21 | (3.9) |
| 25-27 | 20 | (3.3) | 37 | (4.4) | 45 | (4.7) | 29 | (5.1) | 39 | (5.5) | 45 | (5.1) | 52 | (4.9) | 31 | (5.1) |
| 28-30 | 40 | (6.1) | 35 | (5.1) | 56 | (5.5) | 35 | (4.7) | 36 | (5.0) | 40 | (5.7) | 69 | (6.4) | 43 | (4.8) |
| 31-32 | 51 | (7.2) | 38 | (5.5) | 57 | (6.9) | 49 | (5.9) | 55 | (7.2) | 50 | (7.3) | 54 | (7.2) | 61 | (7.1) |
| 34-36 | 63 | (8.1) | 56 | (8.2) | 54 | (7.0) | 44 | (6.9) | 86 | (9.3) | 67 | (9.7) | 61 | (9.0) | 57 | (9.4) |
| 37-39 | 87 | (8.3) | 57 | (8.2) | 65 | (9.0) | 49 | (8.4) | 153 | (13.5) | 87 | (10.9) | 79 | (11.4) | 44 | (7.8) |
| 40-42 | 130 | (11.2) | 82 | (8.4) | 73 | (9.5) | 57 | (9.5) | 212 | (16.3) | 139 | (14.0) | 95 | (12.3) | 52 | (9.5) |
| 43-45 | 149 | (13.7) | 109 | (10.3) | 105 | (10.3) | 74 | (12.5) | 179 | (15.1) | 174 | (15.3) | 128 | (13.6) | 85 | (13.0) |
| 46-48 | 159 | (13.0) | 120 | (12.1) | 122 | (11.1) | 94 | (11.9) | 214 | (15.4) | 190 | (17.5) | 220 | (19.6) | 112 | (14.6) |
| 49-51 | 95 | (14.2) | 140 | (13.0) | 160 | (15.6) | 121 | (13.4) | 144 | (20.9) | 231 | (19.6) | 231 | (22.1) | 186 | (20.3) |
| 52-54 | 154 | (15.1) | 95 | (16.1) | 207 | (18.5) | 125 | (15.6) | 212 | (21.0) | 139 | (23.0) | 286 | (24.9) | 203 | (24.1) |
| 55-57 | 181 | (16.9) | 145 | (16.2) | 109 | (19.0) | 161 | (18.5) | 232 | (20.4) | 188 | (21.2) | 149 | (26.1) | 228 | (24.6) |
| 58-60 | 189 | (21.8) | 192 | (20.4) | 175 | (20.3) | 92 | (19.9) | 216 | (23.3) | 245 | (24.8) | 241 | (29.0) | 120 | (27.5) |
| 61-63 | 175 | (22.0) | 183 | (23.9) | 215 | (23.9) | 135 | (20.6) | 210 | (28.0) | 210 | (25.5) | 309 | (33.0) | 190 | (30.9) |
| 64-66 | 143 | (22.3) | 144 | (20.9) | 198 | (27.8) | 190 | (27.4) | 158 | (25.1) | 192 | (29.3) | 236 | (30.8) | 234 | (33.1) |
| 67-69 | 102 | (20.2) | 129 | (23.9) | 178 | (27.1) | 148 | (28.2) | 139 | (32.9) | 155 | (28.3) | 216 | (34.8) | 175 | (30.8) |
| 70-72 | 124 | (28.4) | 113 | (26.7) | 156 | (31.9) | 150 | (31.6) | 105 | (28.3) | 117 | (32.4) | 182 | (35.5) | 156 | (37.4) |
| 73-75 | 106 | (29.7) | 89 | (25.6) | 108 | (30.0) | 95 | (31.2) | 100 | (29.8) | 88 | (31.9) | 110 | (36.3) | 124 | (37.3) |
| 76-78 | 70 | (27.0) | 52 | (26.1) | 88 | (32.0) | 62 | (33.5) | 70 | (26.0) | 59 | (25.8) | 82 | (36.3) | 64 | (41.6) |
| 79-82 | 25 | (26.3) | 34 | (23.8) | 41 | (37.6) | 37 | (34.9) | 23 | (22.1) | 48 | (33.6) | 45 | (37.8) | 29 | (34.3) |
| 82-85 | 24 | (23.8) | 24 | (23.2) | 36 | (30.0) | 31 | (31.4) | 31 | (20.5) | 38 | (28.9) | 38 | (31.9) | 33 | (33.3) |

Continued

| Residence | Urban(n%) | | | | | | | | Rural(n%) | | | | | | | |
| --- | --- | --- | --- | --- | --- | --- | --- | --- | --- | --- | --- | --- | --- | --- | --- | --- |
| Age/Period | 2010 | | 2013 | | 2016 | | 2019 | | 2010 | | 2013 | | 2016 | | 2019 | |
| 18-21 | 35 | (4.5) | 23 | (3.3) | 28 | (3.4) | 21 | (3.4) | 41 | (3.9) | 28 | (3.1) | 36 | (4.3) | 15 | (2.6) |
| 22-24 | 20 | (3.0) | 29 | (4.1) | 33 | (4.5) | 25 | (4.5) | 34 | (4.1) | 33 | (3.8) | 22 | (3.0) | 11 | (2.3) |
| 25-27 | 23 | (3.4) | 41 | (5.1) | 48 | (4.4) | 40 | (5.9) | 36 | (5.7) | 40 | (4.5) | 49 | (5.2) | 19 | (3.9) |
| 28-30 | 39 | (5.3) | 36 | (5.3) | 84 | (7.5) | 42 | (4.5) | 37 | (5.8) | 39 | (5.5) | 41 | (4.2) | 36 | (5.2) |
| 31-32 | 48 | (6.5) | 47 | (6.7) | 64 | (7.4) | 63 | (6.4) | 58 | (7.9) | 40 | (6.1) | 47 | (6.7) | 47 | (6.6) |
| 34-36 | 76 | (8.9) | 57 | (8.3) | 64 | (8.2) | 58 | (8.4) | 73 | (8.6) | 66 | (9.6) | 51 | (7.6) | 43 | (7.8) |
| 37-39 | 107 | (10.7) | 69 | (9.2) | 74 | (10.0) | 56 | (8.8) | 133 | (11.3) | 75 | (10.0) | 70 | (10.4) | 37 | (7.2) |
| 40-42 | 167 | (14.9) | 116 | (12.3) | 92 | (11.4) | 59 | (9.6) | 175 | (13.0) | 105 | (10.3) | 76 | (10.3) | 50 | (9.3) |
| 43-45 | 122 | (13.1) | 142 | (13.7) | 127 | (13.0) | 85 | (12.8) | 206 | (15.3) | 141 | (12.1) | 106 | (10.8) | 74 | (12.7) |
| 46-48 | 174 | (14.9) | 127 | (15.0) | 166 | (15.2) | 112 | (14.5) | 199 | (13.8) | 184 | (14.9) | 176 | (15.6) | 94 | (11.9) |
| 49-51 | 94 | (14.2) | 172 | (17.1) | 153 | (17.7) | 152 | (16.6) | 145 | (20.8) | 199 | (15.9) | 238 | (19.8) | 156 | (17.1) |
| 52-54 | 167 | (16.8) | 118 | (20.5) | 219 | (21.1) | 128 | (17.9) | 199 | (19.3) | 116 | (18.7) | 274 | (22.3) | 200 | (21.6) |
| 55-57 | 181 | (17.5) | 160 | (19.3) | 124 | (22.3) | 183 | (21.8) | 232 | (19.8) | 173 | (18.1) | 134 | (22.8) | 206 | (21.5) |
| 58-60 | 186 | (22.6) | 211 | (23.7) | 181 | (22.9) | 98 | (21.7) | 219 | (22.6) | 226 | (21.7) | 235 | (26.0) | 115 | (25.5) |
| 61-63 | 184 | (26.3) | 192 | (26.8) | 260 | (30.0) | 154 | (24.9) | 201 | (23.8) | 201 | (23.0) | 264 | (27.3) | 171 | (26.3) |
| 64-66 | 143 | (25.4) | 162 | (26.8) | 228 | (33.4) | 191 | (28.5) | 158 | (22.3) | 174 | (23.6) | 206 | (25.9) | 233 | (31.9) |
| 67-69 | 95 | (24.9) | 132 | (26.5) | 198 | (33.3) | 155 | (30.0) | 146 | (26.7) | 152 | (25.7) | 196 | (28.8) | 168 | (29.2) |
| 70-72 | 112 | (29.8) | 104 | (32.4) | 170 | (35.5) | 159 | (37.6) | 117 | (27.1) | 125 | (27.1) | 168 | (32.2) | 147 | (31.3) |
| 73-75 | 99 | (29.7) | 100 | (32.7) | 100 | (35.6) | 111 | (34.0) | 107 | (29.7) | 77 | (24.3) | 118 | (30.9) | 108 | (34.8) |
| 76-78 | 61 | (22.8) | 61 | (28.3) | 91 | (35.8) | 56 | (36.8) | 79 | (30.4) | 51 | (23.5) | 79 | (32.0) | 71 | (37.5) |
| 79-82 | 26 | (22.8) | 47 | (34.2) | 48 | (39.3) | 44 | (41.4) | 22 | (25.9) | 35 | (23.6) | 38 | (35.8) | 23 | (26.3) |
| 82-85 | 32 | (24.8) | 37 | (28.4) | 43 | (33.6) | 39 | (31.4) | 23 | (18.7) | 25 | (23.9) | 31 | (27.9) | 25 | (34.0) |

| Province | Severe famine provinces | | | | | | | | Mild famine provinces | | | | | | | |
| --- | --- | --- | --- | --- | --- | --- | --- | --- | --- | --- | --- | --- | --- | --- | --- | --- |
| Age/Period | 2010 | | 2013 | | 2016 | | 2019 | | 2010 | | 2013 | | 2016 | | 2019 | |
| 18-21 | 34 | (5.3) | 19 | (2.9) | 22 | (3.1) | 13 | (2.6) | 42 | (3.5) | 32 | (3.4) | 42 | (4.4) | 23 | (3.3) |
| 22-24 | 24 | (4.4) | 21 | (3.3) | 26 | (4.5) | 11 | (2.5) | 30 | (3.1) | 41 | (4.3) | 29 | (3.3) | 25 | (4.1) |
| 25-27 | 19 | (4.2) | 26 | (4.0) | 32 | (4.1) | 19 | (4.4) | 40 | (4.7) | 56 | (5.2) | 65 | (5.2) | 41 | (5.5) |
| 28-30 | 32 | (7.3) | 30 | (5.6) | 41 | (5.5) | 27 | (4.3) | 44 | (4.7) | 45 | (5.3) | 84 | (6.2) | 51 | (5.1) |
| 31-32 | 45 | (9.2) | 32 | (6.9) | 41 | (6.8) | 41 | (7.0) | 61 | (6.2) | 55 | (6.2) | 70 | (7.2) | 68 | (6.2) |
| 34-36 | 59 | (9.7) | 51 | (10.8) | 37 | (7.1) | 38 | (8.2) | 90 | (8.3) | 71 | (8.0) | 78 | (8.4) | 63 | (8.1) |
| 37-39 | 98 | (12.4) | 62 | (10.9) | 67 | (13.2) | 36 | (8.7) | 142 | (10.2) | 82 | (8.8) | 77 | (8.5) | 57 | (7.8) |
| 40-42 | 143 | (16.0) | 87 | (11.6) | 66 | (11.2) | 50 | (12.1) | 199 | (12.7) | 134 | (11.0) | 102 | (10.7) | 59 | (8.0) |
| 43-45 | 140 | (15.9) | 114 | (14.0) | 107 | (13.8) | 66 | (13.8) | 188 | (13.5) | 169 | (12.2) | 126 | (10.6) | 93 | (12.1) |
| 46-48 | 157 | (17.1) | 126 | (15.7) | 153 | (17.5) | 82 | (13.7) | 216 | (12.7) | 184 | (14.5) | 189 | (14.0) | 124 | (12.9) |
| 49-51 | 79 | (21.4) | 133 | (16.2) | 176 | (21.3) | 127 | (18.1) | 160 | (16.2) | 238 | (16.6) | 215 | (17.3) | 180 | (16.1) |
| 52-54 | 109 | (19.0) | 72 | (20.9) | 211 | (24.7) | 149 | (22.8) | 257 | (17.7) | 162 | (19.0) | 282 | (19.9) | 180 | (18.1) |
| 55-57 | 145 | (20.8) | 91 | (17.7) | 82 | (24.1) | 156 | (23.5) | 268 | (17.7) | 242 | (19.0) | 176 | (21.9) | 233 | (20.5) |
| 58-60 | 149 | (26.2) | 143 | (22.8) | 134 | (26.2) | 66 | (24.1) | 256 | (20.9) | 294 | (22.5) | 282 | (23.9) | 147 | (23.4) |
| 61-63 | 131 | (25.2) | 133 | (25.1) | 178 | (29.4) | 109 | (28.9) | 254 | (24.8) | 260 | (24.6) | 346 | (28.1) | 216 | (24.2) |
| 64-66 | 119 | (26.6) | 106 | (22.6) | 168 | (32.4) | 152 | (32.5) | 182 | (22.1) | 230 | (26.3) | 266 | (27.8) | 272 | (29.1) |
| 67-69 | 98 | (28.4) | 104 | (27.5) | 141 | (30.9) | 114 | (30.8) | 143 | (24.6) | 180 | (25.3) | 253 | (30.9) | 209 | (28.9) |
| 70-72 | 80 | (27.8) | 84 | (28.2) | 120 | (32.5) | 111 | (35.8) | 149 | (28.7) | 146 | (30.0) | 218 | (34.5) | 195 | (33.5) |
| 73-75 | 71 | (32.4) | 51 | (23.8) | 94 | (35.7) | 85 | (38.4) | 135 | (28.5) | 126 | (30.8) | 124 | (31.0) | 135 | (32.3) |
| 76-78 | 44 | (27.8) | 27 | (20.2) | 56 | (32.9) | 55 | (44.0) | 96 | (25.9) | 85 | (28.4) | 114 | (34.4) | 71 | (33.2) |
| 79-82 | 13 | (21.3) | 21 | (23.0) | 29 | (43.9) | 21 | (32.6) | 35 | (25.4) | 61 | (31.4) | 57 | (35.2) | 45 | (35.7) |
| 82-85 | 16 | (20.0) | 14 | (18.5) | 20 | (26.0) | 16 | (29.8) | 39 | (22.7) | 48.5 | (29.9) | 54 | (33.3) | 48 | (33.3) |

Continued

TableS2: Prevalence of NCDs by age, period, and birth cohort, 2010-2019 (per 1000 population)

| Median period | Age group | | | | | | | | | | | | | | | | | | | | | | | |
| --- | --- | --- | --- | --- | --- | --- | --- | --- | --- | --- | --- | --- | --- | --- | --- | --- | --- | --- | --- | --- | --- | --- | --- | --- |
|  | 20 | 23 | 26 | 29 | 32 | 35 | 38 | 41 | 44 | 47 | 50 | 53 | 56 | 59 | 62 | 65 | 68 | 71 | 74 | 77 | 80 | 83 | Median Birth cohort |  |
| Male |  |  |  |  |  |  |  |  |  |  |  |  |  |  |  |  |  |  |  |  |  | 237.6 | 1927 |  |
|  |  |  |  |  |  |  |  |  |  |  |  |  |  |  |  |  |  |  |  |  | 263.2 | 231.9 | 1930 |  |
|  |  |  |  |  |  |  |  |  |  |  |  |  |  |  |  |  |  |  |  | 270.3 | 238.4 | 300.0 | 1933 |  |
|  |  |  |  |  |  |  |  |  |  |  |  |  |  |  |  |  |  |  | 296.9 | 260.7 | 376.1 | 314.4 | 1936 |  |
|  |  |  |  |  |  |  |  |  |  |  |  |  |  |  |  |  |  | 283.8 | 256.1 | 320.0 | 349.1 |  | 1939 |  |
|  |  |  |  |  |  |  |  |  |  |  |  |  |  |  |  |  | 202.0 | 266.9 | 300.0 | 335.1 |  |  | 1942 |  |
|  |  |  |  |  |  |  |  |  |  |  |  |  |  |  |  | 222.7 | 238.8 | 319.0 | 312.0 |  |  |  | 1945 |  |
|  |  |  |  |  |  |  |  |  |  |  |  |  |  |  | 220.4 | 209.3 | 271.3 | 315.8 |  |  |  |  | 1948 |  |
|  |  |  |  |  |  |  |  |  |  |  |  |  |  | 218.5 | 239.3 | 278.1 | 282.0 |  |  |  |  |  | 1951 |  |
|  |  |  |  |  |  |  |  |  |  |  |  |  | 169.3 | 203.6 | 239.2 | 274.0 |  |  |  |  |  |  | 1954 |  |
|  |  |  |  |  |  |  |  |  |  |  |  | 151.4 | 161.7 | 203.3 | 206.3 |  |  |  |  |  |  |  | 1957 |  |
|  |  |  |  |  |  |  |  |  |  |  | 142.2 | 161.2 | 189.9 | 199.1 |  |  |  |  |  |  |  |  | 1960 |  |
|  |  |  |  |  |  |  |  |  |  | 129.6 | 130.0 | 184.8 | 184.7 |  |  |  |  |  |  |  |  |  | 1963 |  |
|  |  |  |  |  |  |  |  |  | 136.6 | 121.0 | 156.4 | 156.4 |  |  |  |  |  |  |  |  |  |  | 1966 |  |
|  |  |  |  |  |  |  |  | 111.9 | 102.8 | 111.0 | 134.1 |  |  |  |  |  |  |  |  |  |  |  | 1969 |  |
|  |  |  |  |  |  |  | 83.4 | 84.06 | 103.3 | 118.6 |  |  |  |  |  |  |  |  |  |  |  |  | 1972 |  |
|  |  |  |  |  |  | 81.4 | 81.9 | 95.05 | 125.1 |  |  |  |  |  |  |  |  |  |  |  |  |  | 1975 |  |
|  |  |  |  |  | 72.1 | 82.2 | 89.7 | 94.64 |  |  |  |  |  |  |  |  |  |  |  |  |  |  | 1978 |  |
|  |  |  |  | 60.6 | 55.3 | 69.9 | 84.2 |  |  |  |  |  |  |  |  |  |  |  |  |  |  |  | 1981 |  |
|  |  |  | 33.4 | 50.7 | 69.3 | 69.0 |  |  |  |  |  |  |  |  |  |  |  |  |  |  |  |  | 1984 |  |
|  |  | 33.0 | 44.3 | 54.6 | 58.6 |  |  |  |  |  |  |  |  |  |  |  |  |  |  |  |  |  | 1987 |  |
|  | 43.1 | 37.3 | 46.6 | 47.3 |  |  |  |  |  |  |  |  |  |  |  |  |  |  |  |  |  |  | 1990 |  |
| 2010↗ | 35.9 | 44.7 | 50.6 |  |  |  |  |  |  |  |  |  |  |  |  |  |  |  |  |  |  |  | 1993 |  |
| 2013↗ | 36.9 | 30.0 |  |  |  |  |  |  |  |  |  |  |  |  |  |  |  |  |  |  |  |  | 1996 |  |
| 2016↗ | 29.8 |  |  |  |  |  |  |  |  |  |  |  |  |  |  |  |  |  |  |  |  |  | 1999 |  |
| 2019↗ | |  |  |  |  |  |  |  |  |  |  |  |  |  |  |  |  |  |  |  |  |  |  |  |

| Median period | Age group | | | | | | | | | | | | | | | | | | | | | | |  | |
| --- | --- | --- | --- | --- | --- | --- | --- | --- | --- | --- | --- | --- | --- | --- | --- | --- | --- | --- | --- | --- | --- | --- | --- | --- | --- |
|  | 20 | 23 | 26 | 29 | 32 | 35 | 38 | 41 | 44 | 47 | 50 | 53 | 56 | 59 | 62 | 65 | 68 | 71 | 74 | 77 | 80 | 83 | Median  Birth cohort | |  |
| Female | |  |  |  |  |  |  |  |  |  |  |  |  |  |  |  |  |  |  |  |  | 205.3 | 1927 | |  |
|  |  |  |  |  |  |  |  |  |  |  |  |  |  |  |  |  |  |  |  |  | 221.2 | 287.9 | 1930 | |  |
|  |  |  |  |  |  |  |  |  |  |  |  |  |  |  |  |  |  |  |  | 260.2 | 335.7 | 319.3 | 1933 | |  |
|  |  |  |  |  |  |  |  |  |  |  |  |  |  |  |  |  |  |  | 297.6 | 257.6 | 378.2 | 333.3 | 1936 | |  |
|  |  |  |  |  |  |  |  |  |  |  |  |  |  |  |  |  |  | 283.0 | 319.3 | 362.8 | 343.2 |  | 1939 | |  |
|  |  |  |  |  |  |  |  |  |  |  |  |  |  |  |  |  | 329.4 | 324.1 | 363.0 | 415.6 |  |  | 1942 | |  |
|  |  |  |  |  |  |  |  |  |  |  |  |  |  |  |  | 250.8 | 282.7 | 355.5 | 372.9 |  |  |  | 1945 | |  |
|  |  |  |  |  |  |  |  |  |  |  |  |  |  |  | 280.0 | 293.1 | 348.4 | 374.2 |  |  |  |  | 1948 | |  |
|  |  |  |  |  |  |  |  |  |  |  |  |  |  | 233.0 | 255.2 | 308.5 | 308.1 |  |  |  |  |  | 1951 | |  |
|  |  |  |  |  |  |  |  |  |  |  |  |  | 203.9 | 247.7 | 329.8 | 330.7 |  |  |  |  |  |  | 1954 | |  |
|  |  |  |  |  |  |  |  |  |  |  |  | 210.3 | 211.8 | 290.0 | 309.4 |  |  |  |  |  |  |  | 1957 | |  |
|  |  |  |  |  |  |  |  |  |  |  | 208.7 | 229.9 | 260.9 | 274.6 |  |  |  |  |  |  |  |  | 1960 | |  |
|  |  |  |  |  |  |  |  |  |  | 154.3 | 195.9 | 248.7 | 246.2 |  |  |  |  |  |  |  |  |  | 1963 | |  |
|  |  |  |  |  |  |  |  |  | 151.3 | 175.1 | 221.5 | 240.7 |  |  |  |  |  |  |  |  |  |  | 1966 | |  |
|  |  |  |  |  |  |  |  | 162.7 | 152.8 | 195.9 | 202.7 |  |  |  |  |  |  |  |  |  |  |  | 1969 | |  |
|  |  |  |  |  |  |  | 134.9 | 140.0 | 136.0 | 146.0 |  |  |  |  |  |  |  |  |  |  |  |  | 1972 | |  |
|  |  |  |  |  |  | 93.4 | 108.7 | 122.7 | 129.5 |  |  |  |  |  |  |  |  |  |  |  |  |  | 1975 | |  |
|  |  |  |  |  | 71.5 | 96.9 | 114.3 | 95.0 |  |  |  |  |  |  |  |  |  |  |  |  |  |  | 1978 | |  |
|  |  |  |  | 50.4 | 73.1 | 90.0 | 78.2 |  |  |  |  |  |  |  |  |  |  |  |  |  |  |  | 1981 | |  |
|  |  |  | 55.1 | 57.1 | 71.9 | 94.0 |  |  |  |  |  |  |  |  |  |  |  |  |  |  |  |  | 1984 | |  |
|  |  | 38.3 | 50.6 | 64.4 | 70.8 |  |  |  |  |  |  |  |  |  |  |  |  |  |  |  |  |  | 1987 | |  |
|  | 39.6 | 40.5 | 48.6 | 48.0 |  |  |  |  |  |  |  |  |  |  |  |  |  |  |  |  |  |  | 1990 | |  |
| 2010↗ | 28.3 | 31.1 | 51.3 |  |  |  |  |  |  |  |  |  |  |  |  |  |  |  |  |  |  |  | 1993 | |  |
| 2013↗ | 39.8 | 38.6 |  |  |  |  |  |  |  |  |  |  |  |  |  |  |  |  |  |  |  |  | 1996 | |  |
| 2016↗ | 29.9 |  |  |  |  |  |  |  |  |  |  |  |  |  |  |  |  |  |  |  |  |  | 1999 | |  |
| 2019↗ |  |  |  |  |  |  |  |  |  |  |  |  |  |  |  |  |  |  |  |  |  |  |  | |  |

Continued

|  | Age group | | | | | | | | | | | | | | | | | | | | | | |  | |
| --- | --- | --- | --- | --- | --- | --- | --- | --- | --- | --- | --- | --- | --- | --- | --- | --- | --- | --- | --- | --- | --- | --- | --- | --- | --- |
| Median period | 20 | 23 | 26 | 29 | 32 | 35 | 38 | 41 | 44 | 47 | 50 | 53 | 56 | 59 | 62 | 65 | 68 | 71 | 74 | 77 | 80 | 83 | Median  Birth cohort | |  |
| Urban |  |  |  |  |  |  |  |  |  |  |  |  |  |  |  |  |  |  |  |  |  | 248.1 | 1927 | |  |
|  |  |  |  |  |  |  |  |  |  |  |  |  |  |  |  |  |  |  |  |  | 228.1 | 283.5 | 1930 | |  |
|  |  |  |  |  |  |  |  |  |  |  |  |  |  |  |  |  |  |  |  | 227.6 | 341.8 | 335.9 | 1933 | |  |
|  |  |  |  |  |  |  |  |  |  |  |  |  |  |  |  |  |  |  | 297.3 | 282.7 | 393.4 | 314.3 | 1936 | |  |
|  |  |  |  |  |  |  |  |  |  |  |  |  |  |  |  |  |  | 297.9 | 326.8 | 358.3 | 414.3 |  | 1939 | |  |
|  |  |  |  |  |  |  |  |  |  |  |  |  |  |  |  |  | 249.3 | 324.5 | 355.9 | 367.5 |  |  | 1942 | |  |
|  |  |  |  |  |  |  |  |  |  |  |  |  |  |  |  | 254.0 | 265.1 | 354.9 | 340.0 |  |  |  | 1945 | |  |
|  |  |  |  |  |  |  |  |  |  |  |  |  |  |  | 263.2 | 268.2 | 332.8 | 375.9 |  |  |  |  | 1948 | |  |
|  |  |  |  |  |  |  |  |  |  |  |  |  |  | 226.3 | 268.4 | 334.3 | 300.0 |  |  |  |  |  | 1951 | |  |
|  |  |  |  |  |  |  |  |  |  |  |  |  | 174.7 | 237.5 | 299.5 | 285.3 |  |  |  |  |  |  | 1954 | |  |
|  |  |  |  |  |  |  |  |  |  |  |  | 168.3 | 193.0 | 229.4 | 249.2 |  |  |  |  |  |  |  | 1957 | |  |
|  |  |  |  |  |  |  |  |  |  |  | 142.2 | 205.0 | 222.6 | 216.9 |  |  |  |  |  |  |  |  | 1960 | |  |
|  |  |  |  |  |  |  |  |  |  | 148.8 | 171.2 | 210.8 | 218.4 |  |  |  |  |  |  |  |  |  | 1963 | |  |
|  |  |  |  |  |  |  |  |  | 131.5 | 149.8 | 177.5 | 178.6 |  |  |  |  |  |  |  |  |  |  | 1966 | |  |
|  |  |  |  |  |  |  |  | 149.0 | 137.4 | 152.2 | 166.1 |  |  |  |  |  |  |  |  |  |  |  | 1969 | |  |
|  |  |  |  |  |  |  | 106.7 | 122.6 | 129.9 | 145.0 |  |  |  |  |  |  |  |  |  |  |  |  | 1972 | |  |
|  |  |  |  |  |  | 89.4 | 92.3 | 114.4 | 127.6 |  |  |  |  |  |  |  |  |  |  |  |  |  | 1975 | |  |
|  |  |  |  |  | 64.9 | 83.3 | 99.7 | 96.5 |  |  |  |  |  |  |  |  |  |  |  |  |  |  | 1978 | |  |
|  |  |  |  | 52.9 | 67.0 | 82.4 | 88.4 |  |  |  |  |  |  |  |  |  |  |  |  |  |  |  | 1981 | |  |
|  |  |  | 34.1 | 53.2 | 73.6 | 83.9 |  |  |  |  |  |  |  |  |  |  |  |  |  |  |  |  | 1984 | |  |
|  |  | 29.6 | 50.6 | 75.2 | 64.3 |  |  |  |  |  |  |  |  |  |  |  |  |  |  |  |  |  | 1987 | |  |
|  | 44.7 | 40.8 | 44.1 | 44.6 |  |  |  |  |  |  |  |  |  |  |  |  |  |  |  |  |  |  | 1990 | |  |
| 2010↗ | 33.2 | 44.8 | 59.2 |  |  |  |  |  |  |  |  |  |  |  |  |  |  |  |  |  |  |  | 1993 | |  |
| 2013↗ | 33.6 | 44.5 |  |  |  |  |  |  |  |  |  |  |  |  |  |  |  |  |  |  |  |  | 1996 | |  |
| 2016↗ | 33.8 |  |  |  |  |  |  |  |  |  |  |  |  |  |  |  |  |  |  |  |  |  | 1999 | |  |
| 2019↗ |  |  |  |  |  |  |  |  |  |  |  |  |  |  |  |  |  |  |  |  |  |  |  | |  |

Continued

Continued

|  | Age group | | | | | | | | | | | | | | | | | | | | | |  |
| --- | --- | --- | --- | --- | --- | --- | --- | --- | --- | --- | --- | --- | --- | --- | --- | --- | --- | --- | --- | --- | --- | --- | --- |
| Median period | 20 | 23 | 26 | 29 | 32 | 35 | 38 | 41 | 44 | 47 | 50 | 53 | 56 | 59 | 62 | 65 | 68 | 71 | 74 | 77 | 80 | 83 | Median  Birth cohort |
| Rural |  |  |  |  |  |  |  |  |  |  |  |  |  |  |  |  |  |  |  |  |  | 187.0 | 1927 |
|  |  |  |  |  |  |  |  |  |  |  |  |  |  |  |  |  |  |  |  |  | 258.8 | 239.2 | 1930 |
|  |  |  |  |  |  |  |  |  |  |  |  |  |  |  |  |  |  |  |  | 303.8 | 236.3 | 279.3 | 1933 |
|  |  |  |  |  |  |  |  |  |  |  |  |  |  |  |  |  |  |  | 297.2 | 235.4 | 358.5 | 340.3 | 1936 |
|  |  |  |  |  |  |  |  |  |  |  |  |  |  |  |  |  |  | 270.8 | 242.9 | 319.8 | 263.2 |  | 1939 |
|  |  |  |  |  |  |  |  |  |  |  |  |  |  |  |  |  | 267.4 | 271.4 | 308.9 | 375.0 |  |  | 1942 |
|  |  |  |  |  |  |  |  |  |  |  |  |  |  |  |  | 222.8 | 257.4 | 321.8 | 347.8 |  |  |  | 1945 |
|  |  |  |  |  |  |  |  |  |  |  |  |  |  |  | 237.9 | 235.6 | 287.8 | 313.4 |  |  |  |  | 1948 |
|  |  |  |  |  |  |  |  |  |  |  |  |  |  | 225.8 | 230.5 | 259.1 | 291.7 |  |  |  |  |  | 1951 |
|  |  |  |  |  |  |  |  |  |  |  |  |  | 198.1 | 216.6 | 272.7 | 318.5 |  |  |  |  |  |  | 1954 |
|  |  |  |  |  |  |  |  |  |  |  |  | 192.6 | 181.2 | 260.2 | 262.7 |  |  |  |  |  |  |  | 1957 |
|  |  |  |  |  |  |  |  |  |  |  | 208.0 | 187.3 | 227.9 | 254.7 |  |  |  |  |  |  |  |  | 1960 |
|  |  |  |  |  |  |  |  |  |  | 137.7 | 159.0 | 222.6 | 214.6 |  |  |  |  |  |  |  |  |  | 1963 |
|  |  |  |  |  |  |  |  |  | 153.0 | 148.9 | 197.7 | 216.0 |  |  |  |  |  |  |  |  |  |  | 1966 |
|  |  |  |  |  |  |  |  | 130.2 | 120.9 | 155.6 | 171.3 |  |  |  |  |  |  |  |  |  |  |  | 1969 |
|  |  |  |  |  |  |  | 113.3 | 102.8 | 108.3 | 119.4 |  |  |  |  |  |  |  |  |  |  |  |  | 1972 |
|  |  |  |  |  |  | 86.4 | 100.1 | 103.0 | 127.1 |  |  |  |  |  |  |  |  |  |  |  |  |  | 1975 |
|  |  |  |  |  | 78.8 | 95.8 | 103.9 | 92.9 |  |  |  |  |  |  |  |  |  |  |  |  |  |  | 1978 |
|  |  |  |  | 58.1 | 61.2 | 75.7 | 72.5 |  |  |  |  |  |  |  |  |  |  |  |  |  |  |  | 1981 |
|  |  |  | 56.9 | 54.7 | 66.7 | 77.6 |  |  |  |  |  |  |  |  |  |  |  |  |  |  |  |  | 1984 |
|  |  | 40.9 | 44.8 | 41.8 | 65.5 |  |  |  |  |  |  |  |  |  |  |  |  |  |  |  |  |  | 1987 |
|  | 38.8 | 37.7 | 51.8 | 51.9 |  |  |  |  |  |  |  |  |  |  |  |  |  |  |  |  |  |  | 1990 |
| 2010↗ | 31.1 | 30.2 | 39.4 |  |  |  |  |  |  |  |  |  |  |  |  |  |  |  |  |  |  |  | 1993 |
| 2013↗ | 43.1 | 22.6 |  |  |  |  |  |  |  |  |  |  |  |  |  |  |  |  |  |  |  |  | 1996 |
| 2016↗ | 25.7 |  |  |  |  |  |  |  |  |  |  |  |  |  |  |  |  |  |  |  |  |  | 1999 |
| 2019↗ | |  |  |  |  |  |  |  |  |  |  |  |  |  |  |  |  |  |  |  |  |  |  |

Continued

|  | Age group | | | | | | | | | | | | | | | | | | | | | | |  | |
| --- | --- | --- | --- | --- | --- | --- | --- | --- | --- | --- | --- | --- | --- | --- | --- | --- | --- | --- | --- | --- | --- | --- | --- | --- | --- |
| Median Period | 20 | 23 | 26 | 29 | 32 | 35 | 38 | 41 | 44 | 47 | 50 | 53 | 56 | 59 | 62 | 65 | 68 | 71 | 74 | 77 | 80 | 83 | Median Birth cohort | |  |
| Severe famine provinces | | | |  |  |  |  |  |  |  |  |  |  |  |  |  |  |  |  |  |  | 200.0 | 1927 | |  |
|  |  |  |  |  |  |  |  |  |  |  |  |  |  |  |  |  |  |  |  |  | 213.1 | 184.9 | 1930 | |  |
|  |  |  |  |  |  |  |  |  |  |  |  |  |  |  |  |  |  |  |  | 278.5 | 230.3 | 259.7 | 1933 | |  |
|  |  |  |  |  |  |  |  |  |  |  |  |  |  |  |  |  |  |  | 324.2 | 202.3 | 439.4 | 298.1 | 1936 | |  |
|  |  |  |  |  |  |  |  |  |  |  |  |  |  |  |  |  |  | 277.8 | 237.8 | 329.4 | 325.6 |  | 1939 | |  |
|  |  |  |  |  |  |  |  |  |  |  |  |  |  |  |  |  | 284.1 | 282.1 | 357.4 | 440.0 |  |  | 1942 | |  |
|  |  |  |  |  |  |  |  |  |  |  |  |  |  |  |  | 266.2 | 275.3 | 325.2 | 384.1 |  |  |  | 1945 | |  |
|  |  |  |  |  |  |  |  |  |  |  |  |  |  |  | 251.9 | 225.9 | 308.5 | 358.2 |  |  |  |  | 1948 | |  |
|  |  |  |  |  |  |  |  |  |  |  |  |  |  | 261.9 | 250.7 | 323.7 | 308.1 |  |  |  |  |  | 1951 | |  |
|  |  |  |  |  |  |  |  |  |  |  |  |  | 208.0 | 228.4 | 293.7 | 325.5 |  |  |  |  |  |  | 1954 | |  |
|  |  |  |  |  |  |  |  |  |  |  |  | 189.9 | 177.4 | 261.7 | 288.9 |  |  |  |  |  |  |  | 1957 | |  |
|  |  |  |  |  |  |  |  |  |  |  | 213.5 | 209.4 | 241.2 | 240.8 |  |  |  |  |  |  |  |  | 1960 | |  |
|  |  |  |  |  |  |  |  |  |  | 170.8 | 162.0 | 247.4 | 234.9 |  |  |  |  |  |  |  |  |  | 1963 | |  |
|  |  |  |  |  |  |  |  |  | 159.3 | 156.8 | 213.3 | 228.3 |  |  |  |  |  |  |  |  |  |  | 1966 | |  |
|  |  |  |  |  |  |  |  | 160.0 | 140.2 | 174.9 | 181.0 |  |  |  |  |  |  |  |  |  |  |  | 1969 | |  |
|  |  |  |  |  |  |  | 124.1 | 116.2 | 138.4 | 137.1 |  |  |  |  |  |  |  |  |  |  |  |  | 1972 | |  |
|  |  |  |  |  |  | 96.7 | 109.4 | 112.4 | 138.3 |  |  |  |  |  |  |  |  |  |  |  |  |  | 1975 | |  |
|  |  |  |  |  | 92.4 | 108.1 | 131.9 | 120.7 |  |  |  |  |  |  |  |  |  |  |  |  |  |  | 1978 | |  |
|  |  |  |  | 72.9 | 69.4 | 71.4 | 87.4 |  |  |  |  |  |  |  |  |  |  |  |  |  |  |  | 1981 | |  |
|  |  |  | 41.8 | 56.1 | 68.4 | 81.9 |  |  |  |  |  |  |  |  |  |  |  |  |  |  |  |  | 1984 | |  |
|  |  | 44.0 | 40.4 | 55.2 | 69.8 |  |  |  |  |  |  |  |  |  |  |  |  |  |  |  |  |  | 1987 | |  |
|  | 53,1 | 32.8 | 41.3 | 43.0 |  |  |  |  |  |  |  |  |  |  |  |  |  |  |  |  |  |  | 1990 | |  |
| 2010↗ | 28.5 | 44.6 | 44.2 |  |  |  |  |  |  |  |  |  |  |  |  |  |  |  |  |  |  |  | 1993 | |  |
| 2013↗ | 31.0 | 25.4 |  |  |  |  |  |  |  |  |  |  |  |  |  |  |  |  |  |  |  |  | 1996 | |  |
| 2016↗ | 25.7 |  |  |  |  |  |  |  |  |  |  |  |  |  |  |  |  |  |  |  |  |  | 1999 | |  |
| 2019↗ |  |  |  |  |  |  |  |  |  |  |  |  |  |  |  |  |  |  |  |  |  |  |  | |  |

Continued

|  | Age group | | | | | | | | | | | | | | | | | | | | | |  |
| --- | --- | --- | --- | --- | --- | --- | --- | --- | --- | --- | --- | --- | --- | --- | --- | --- | --- | --- | --- | --- | --- | --- | --- |
| Median Period | 20 | 23 | 26 | 29 | 32 | 35 | 38 | 41 | 44 | 47 | 50 | 53 | 56 | 59 | 62 | 65 | 68 | 71 | 74 | 77 | 80 | 83 | Median  Birth cohort |
| Mild famine provinces | | | |  |  |  |  |  |  |  |  |  |  |  |  |  |  |  |  |  |  | 226.7 | 1927 |
|  |  |  |  |  |  |  |  |  |  |  |  |  |  |  |  |  |  |  |  |  | 253.6 | 299.4 | 1930 |
|  |  |  |  |  |  |  |  |  |  |  |  |  |  |  |  |  |  |  |  | 259.5 | 313.6 | 333.3 | 1933 |
|  |  |  |  |  |  |  |  |  |  |  |  |  |  |  |  |  |  |  | 284.8 | 284.0 | 351.9 | 333.3 | 1936 |
|  |  |  |  |  |  |  |  |  |  |  |  |  |  |  |  |  |  | 286.5 | 308.4 | 344.4 | 357.1 |  | 1939 |
|  |  |  |  |  |  |  |  |  |  |  |  |  |  |  |  |  | 245.7 | 300.0 | 310.0 | 331.8 |  |  | 1942 |
|  |  |  |  |  |  |  |  |  |  |  |  |  |  |  |  | 220.6 | 253.4 | 344.9 | 322.5 |  |  |  | 1945 |
|  |  |  |  |  |  |  |  |  |  |  |  |  |  |  | 248.0 | 263.3 | 308.9 | 335.1 |  |  |  |  | 1948 |
|  |  |  |  |  |  |  |  |  |  |  |  |  |  | 209.3 | 246.0 | 277.7 | 289.2 |  |  |  |  |  | 1951 |
|  |  |  |  |  |  |  |  |  |  |  |  |  | 177.5 | 225.1 | 281.3 | 291.2 |  |  |  |  |  |  | 1954 |
|  |  |  |  |  |  |  |  |  |  |  |  | 177.1 | 190.4 | 239.0 | 242.3 |  |  |  |  |  |  |  | 1957 |
|  |  |  |  |  |  |  |  |  |  |  | 161.9 | 190.5 | 218.6 | 233.7 |  |  |  |  |  |  |  |  | 1960 |
|  |  |  |  |  |  |  |  |  |  | 127.4 | 165.8 | 199.0 | 205.5 |  |  |  |  |  |  |  |  |  | 1963 |
|  |  |  |  |  |  |  |  |  | 134.8 | 144.5 | 173.2 | 180.9 |  |  |  |  |  |  |  |  |  |  | 1966 |
|  |  |  |  |  |  |  |  | 126.7 | 121.8 | 140.3 | 160.9 |  |  |  |  |  |  |  |  |  |  |  | 1969 |
|  |  |  |  |  |  |  | 102.4 | 109.9 | 106.4 | 128.9 |  |  |  |  |  |  |  |  |  |  |  |  | 1972 |
|  |  |  |  |  |  | 82.9 | 88.2 | 106.8 | 120.7 |  |  |  |  |  |  |  |  |  |  |  |  |  | 1975 |
|  |  |  |  |  | 61.7 | 79.8 | 84.8 | 80.3 |  |  |  |  |  |  |  |  |  |  |  |  |  |  | 1978 |
|  |  |  |  | 47.1 | 61.5 | 83.6 | 77.8 |  |  |  |  |  |  |  |  |  |  |  |  |  |  |  | 1981 |
|  |  |  | 46.9 | 52.6 | 71.8 | 80.6 |  |  |  |  |  |  |  |  |  |  |  |  |  |  |  |  | 1984 |
|  |  | 31.2 | 51.8 | 62.0 | 62.2 |  |  |  |  |  |  |  |  |  |  |  |  |  |  |  |  |  | 1987 |
|  | 35.0 | 43.1 | 51.6 | 50.7 |  |  |  |  |  |  |  |  |  |  |  |  |  |  |  |  |  |  | 1990 |
| 2010↗ | 34.4 | 32.9 | 54.8 |  |  |  |  |  |  |  |  |  |  |  |  |  |  |  |  |  |  |  | 1993 |
| 2013↗ | 43.8 | 40.7 |  |  |  |  |  |  |  |  |  |  |  |  |  |  |  |  |  |  |  |  | 1996 |
| 2016↗ | 32.9 |  |  |  |  |  |  |  |  |  |  |  |  |  |  |  |  |  |  |  |  |  | 1999 |
| 2019↗ | |  |  |  |  |  |  |  |  |  |  |  |  |  |  |  |  |  |  |  |  |  |  |
